# Cytotoxic and antioxidant properties of active principals isolated from water hyacinth against four cancer cells lines

**DOI:** 10.1186/1472-6882-14-397

**Published:** 2014-10-14

**Authors:** Ahmed M Aboul-Enein, Sanaa MM Shanab, Emad A Shalaby, Malak M Zahran, David A Lightfoot, Hany A El-Shemy

**Affiliations:** Department of Biochemistry, Faculty of Agriculture, Cairo University, 12613 Giza, Egypt; Department of Botany and Microbiology, Faculty of Science, Cairo University, 12613 Giza, Egypt; Department of Cell Biology, National Research Center (NRC), Dokki, Giza, Egypt; Genomics Core-Facility, Southern Illinois University at Carbondale, Carbondle, IL 62901 USA

**Keywords:** *Eichhornia crassipes*, Cytotoxicity, Anticancer, Antioxidant, Active ingredients

## Abstract

**Background:**

*Eichhornia crassipes* (Mart) solms is an invasive macrophyte causing serious problems to the network of irrigation and drainage canals in the Nile Delta region. The present study aim to evaluate the potential anticancer and antioxidant activities of *Eichhornia crassipes* crude extract and its pure compounds.

**Methods:**

The macrophyte was collected from El-Zomor canal, River Nile (Egypt), cleaned, air dried, grinded then extracted with methanol (crude extract). The extract was fractionated using pre-coated silica gel plates (TLC F_254_) with hexane/ethyl acetate (8.5: 1.5 v/v) as mobile phase. Nine fractions were separated (A-I) then scratched, eluted with the same mobile phase, filtered and the separated fractions were determined and identified using spectroscopic methods (Mass spectrum (MS), Infra red (IR) and Proton H-Nuclear magnetic resonance (H-NMR). Both the crude extract and its nine identified compounds were tested for their antioxidant (using 2, 2 diphenyl-1-picrylhydrazyl (DPPH), 2, 2′- azino-bis {ethylbenzthiazoline-6-sulfonic acid (ABTS^.^)} methods) and anticancer activity (using MCF-7, HeLa, Hep.G2 and EACC cell lines).

**Results:**

The antioxidant and anticancer activities of the crude extract exhibited the highest effect while the compounds showed variable effects which depend on the type of compound and cancer cell line. The antioxidant activity of the crude extract exhibited the highest followed in descending order by compounds D, E, G and H respectively. Concerning the anticancer potency, the crude extract showed also the highest effect while the identified compounds (A, B, C, D, E, F, G, H and I) showed variable anticancer activities against the four different cell lines. In addition, Compound I exhibited the most potent anticancer activity against HepG2 cell line while compound D exhibited high anticancer activity against HeLa cells and EACC. The results revealed the presence of different compounds (Alkaloids and terpenoids) with variable antioxidant and anticancer activities which elicited an auto-augmentation in the crude extract leading to its greatest activities. The action of the identified anticancer compounds on DNA fragmentation was studied.

**Conclusion:**

The study illustrated the potential of *Eichhornia* as a valuable resource for natural compounds of desirable medicinal properties (e.g. antioxidants and anticancer).

## Background

The role of plants in maintaining human health is well documented [1]. Plants are used medicinally in different countries and are a source of many potent plus powerful drugs [2]. The active principles of many drugs found in plants were identified from secondary metabolites [3]. Amongst these active phyto-molecules (at least 12,000) about only 10% were chemically identified [4]. The medicinally useful bioactive constituents were belonged to alkaloids, flavonoids, phenolics, essential oils and polyphenols [5]. Alkaloids were play an important metabolic role and control development in living systems [6]. These chemical substances were known to carry out important medicinal roles in human body. They were also involved in protective function in animals and used as medicine especially the steroidal alkaloids [7].

*Eichhornia crassipes* (Mart) Solms, commonly known as water hyacinth, is warm aquatic plant belonging to the family Pontideraceae. Water hyacinth was listed as one of the most productive plants on earth and considered the world’s worst aquatic plant [3]. It originated in the state of Amazone, Brazil, spread to other regions of South America and was carried by humans to tropical and subtropical regions. It invaded Africa and causes extremely serious ecological, economical and social problems in the region between 40 degree north and 45 degree south [8]. The dense mats of water hyacinth cover lakes and rivers, blocking water ways and interfering with the water transport of agriculture products, tourism activities and irrigation of agriculture fields. Its vast growth consumes huge quantity of nutrients favoring and its growth over other aquatic species [9, 10]. Decomposition of dead individuals released nutrients to water leading to deterioration of water quality, clean drinking water which threatened human health [9, 10]. In summer season where temperature exceeded 35°C and high solar radiation with long light duration, *Eichhornia* grows faster with rapid spread in the Egyptian water bodies and serious problems occurred [9, 10].

Epidemiological studies reveal that, numbers of diseases including cancer, diabetes, Alzhimer, coronary heart disease and aging have been found to be associated with oxidative stress and reactive oxygen species (ROS) [11] which damage biomolecules and lead to numerous disease conditions [12]. Dietary antioxidants may reduce the risks of these diseases and improve general human health [13, 14]. Besides their effective role in prevention of diseases, antioxidants were also used as food additives to improve quality of food products by avoiding offensive flavors [15]. Naturally produced antioxidants including flavonoids, tannins, coumarins, xanthones, phenolics, terpenoids were recorded in various plant products (fruits, leaves, seeds and oils) and organisms [9, 10].

Recently, considerable attention has been given to harvest *E. crassipes* for practical uses as economical sources in many parts of the world. Though, very few pharmacological studies on this plant have been reported in literature and are only used as therapeutic properties in folkloric application [16]. Our previous studies showed promising antimicrobial and anti-algal activities of water hyacinth extract and some of its identified compounds [17] encouraged us to go further for other biological effects of this plant. Therefore, the present study aimed to isolate and evaluate the potentially active substances or fractions with potential pharmacological activity of water hyacinth. This investigation was carried out on crude extract of this weed as well as its fractions for potential anticancer and antioxidant activities. The fractions were chemically identified and their action on cancer cells was elucidated.

## Methods

### Chemicals and drugs

Pure hexane, chloroform, ethanol, ether, acetone, methanol and acetic acid were purchased from E. Merck Co. (Darmstadt, Germany). Sulfarhodamine, 2, 2 diphenyl-1-picrylhydrazyl (DPPH), 2, 2′- azino-bis (ethylbenzthiazoline-6-sulfonic acid (ABTS^.+^) were purchased from Sigma-Aldrich (St. Louis, MO, USA). Trichloroacetic acid and other materials were of the highest available commercial grade.

### Collection and extraction of water hyacinth

*E. crassipes* (Mart) Solms was collected during spring season (2010) from the River Nile at El-Zomor canal (Giza), Egypt and was identified by Dr. Sanaa M. M. Shanab, Professor of Phycology, Botany and Microbiology Department, Faculty of Science, Cairo University. This species has been deposited in a scientifically available herbarium (Botany and Microbiology Department, Faculty of Science, Cairo University).

It was cleaned from any debris, washed several times with tap and distilled water then with sterilized water. The samples were air dried (and/or lyophilized), grinded and then stored at -20°C until use. A known weight (500 g) of the macrophyte was extracted three times (Using 5 liter solvents with 24 h intervals) with methanol at 25°C in the dark. The solvent of the combined extracts was evaporated, and solids concentrated using rotary evaporator at 40°C and weighted.

### Fractionation of the crude extract

Using pre-coated TLC F_254_ plates, a solution of the crude methanolic extract (Ten grams) containing a mixture of compounds is applied to the layer of adsorbent, near one edge, as a thin line. The TLC plate is dropped vertically in a closed container (developing chamber), with the edge to which the spot was applied down. The solvents mixture, which is in the bottom of the container, travels up the layer of adsorbent by capillary action, passes over the spot and, as it continues up, crude extract was fractionated using different solvent systems as mobile phase and the suitable one was selected (hexane/ethyl acetate). In this concern, different combinations of hexane/ethyl acetate solvents (9:1, 8.5:1.5, 8:2, 7:3 and 5:5) as the mobile phase were prepared and tested. The compounds were moved using hexane/ethyl acetate (8.5:1.5, v/v), as the best solvent The separated nine fractions (A-I) by the mixture, were scratched and eluted with the same mobile phase then filtered, evaporated (using nitrogen gas), weighed and used for chemical identification and in bioassays experiments [17].

### Instrumental analysis

Potent fractions were chosen for further identifications using the chromatographic and spectroscopic methods as follows:

a- **Mass spectroscopic (MS) analyses of potent fractions**

The biologically potent fractions of *E. crassipes* were analyzed by Mass spectra (MS). The mass spectrum was scanned over the 40 to 500 m/z range with an ionizing voltage of 70 eV and identification was based on standard mass library of National Institute of Standards and Technology (NIST Version 2.0) to detect the possible fraction structure.

b- **Fourier transformed infra red (FTIR) spectra**

A Perkin Elmer (Waltham, Massachusetts, USA) was used to obtain Fourier transformed infrared (FTIR) spectra (System, 2000) of the fractions.

c- **Proton nuclear magnetic resonance Spectra (**^**1**^**H NMR)**

The identification of fractionated compounds was confirmed by carrying out ^1^H-NMR analysis using NMR Joel GIM, EX 270 (400 Hz).

### Assessment of antioxidant activity

Two methods were used for antioxidant evaluation of crude extract as well as its active ingredients as follows:

a- **DPPH method**

The 2, 2 diphenyl-1-picrylhydrazyl (DPPH) tests were carried out for evaluation of antioxidant activity of samples as described by Burits and Bucar [18]. One mL of *Eichhornia* crude extract and fractions at different concentration (From 5 to 500 μg/mL) was mixed with 1 mL DPPH reagent [0.002% (w/v)/ methanol solution]. After an incubation period (30 min), the absorbance was measured at 517 nm. Extract concentration providing 50% inhibition (IC_50_) was calculated from the graph plotting inhibition percentages against extract concentrations compared with Butylated hydroxy anisole (BHA) and Butylated hydroxy toluene (BHT) as synthetic antioxidant and Ascorbic acid (Vit. C) as natural antioxidant standard.


Where: **Ac** was the absorbance of methanolic DPPH solution (control) **and At** was the absorbance of the extract sample.

b- **ABTS**^**+**^**radical cation scavenging assay**

This assay was based on the ability of different fractions to scavenge 2, 2′- azino-bis (ethylbenzthiazoline-6-sulfonic acid (ABTS^.+^) radical cation in comparison to a standard ( BHA, BHT and Ascorbic acid). The radical cation was prepared by mixing a 7 mM ABTS stock solution with 2.45 mM potassium persulfate (1/1, v/v) and leaving the mixture for 4-16 h until the reaction was completed and the absorbance was stable. The ABTS^.+^ solution was diluted with ethanol to an absorbance of 0.700 ± 0.05 at 734 nm for measurements. The photometric Assay was conducted on 0.9 mL of ABTS^+^ solution and 0.1 mL of tested samples (in MeOH solution) and mixed for 45 s; measurements were taken immediately at 734 nm after 1 min. The antioxidant activity (E) of the tested samples was calculated by determining the decrease in absorbance at different concentrations by using the following equation:


Where **At** and **Ac** are the respective absorbance of tested sample and ABTS^+^, [19]. Extracts concentration providing 50% inhibition (IC_50_) was calculated from the graph plotting inhibition percentages against extract concentration.

### Assessment of anticancer activity

a- **Cell culture**

Human hepatocellular cancer cell line (HepG-2), breast cancer cell line (MCF-7) and cervix cancer cell line (HeLa) were obtained from the Vacsera (Giza, Egypt). Cells were maintained in RPMI-1640 supplemented with 100 μg/mL streptomycin, 100 units/mL penicillin and 10% heat-inactivated fetal bovine serum in a humidified, 5% (v/v) CO_2_ atmosphere at 37°C [20]. Ehrlich Ascites Carcinoma Cells, EACC were obtained from National Cancer Institute, NCI, (Cairo, Egypt) and cell line was maintained in female albino mice by i.p transplantation of 0.2 mL ascites which contains about 10^6^ of tumor cells in a liquid form and cells were taken for study after 15-20 days of tumor transplantation.

b- **Cytotoxicity assays**

**1-Against solid tumor cell line**

The cytotoxicity of crude extract and the fractionated compounds were tested against HeLa, HepG-2 and MCF-7 cells by SRB assay as previously described [21] or by neutral red assay [22]. Exponentially growing cells were collected using 0.25% Trypsin-EDTA and plated in 96-well plates at 1000-2000 cells/well. Cells were exposed to each test compound for 72 h and subsequently fixed with TCA (10%) for 1 h at 4°C. After several washings, cells were exposed to 0.4% SRB {sulforhodamine B (SRB), 2-(3-diethylamino-6-diethylazaniumylidene-xanthen-9-yl)-5-sulfo-benzenesulfonate} solution for 10 min in dark place and subsequently washed with 1% glacial acetic acid. After drying overnight, Tris-HCl was used to dissolve the SRB-stained cells and color intensity was measured at 540 nm. Doxorubicin (DOX) purchased from Sigma Chemical Co. (St. Louis, MO, USA) was used as anticancer standard.

### Data analysis

The dose response curve of compounds was analyzed using E_max_ model (Eq. 1).
1

Where R is the residual unaffected fraction (the resistance fraction), [D] is the drug concentration used, K_d_ is the drug concentration that produces a 50% reduction of the maximum inhibition rate and m is a Hill-type coefficient. IC_50_ was defined as the drug concentration required to reduce fluorescence to 50% of that of the control (i.e., K_d_ = IC_50_ when R = 0 and E_max_ =100-R) [22].

### 2-Against liquid tumor cell line

The cytotoxicity of crude extract and its pure fractions was also tested against EACC by viability trypan blue exclusion test according to previous report [20]. In details; A line of Ehrlich Ascites Carcinoma resistant to endoxan has been used. The parent line was first supplied through the coursty of Dr. G. Klein, Amestrdam, Holland. The tumor line is maintained in the National Cancer Institute, Egypt in Female Swiss Albino Mice by weekly transplantation of 2.5×10^6^ cells were centrifuged at 1000 × g for 5 min at 4°C. The pellets were washed with saline (0.9% NaCl) then the needed number of cells was prepared by suspending the cells in the appropriate volume of saline. The viability percentage (V%) of tumor cells was measured after incubation with the tested extract and fractions as well as DMSO as control. Two mL of cells (4×10^6^ cells) were transferred into a set of tubes, then different samples at different concentrations (5 to 500 μg/mL) were added into the propitiate tubes as well as DMSO. The tubes were incubated at 37°C for 2 h. Then, in a test tube containing 80 μl saline and 10 μl trypan blue, 10 μl of cell suspension were added and mixed then the number of living cells was calculated under microscope using a hemocytometer slide.

### DNA extraction and electrophoresis

DNA of cancer cell lines before and after incubation with crude plant extracts and each of the active identified ingredients was extracted using AXYGEN Biosciences (USA) extracted kit. The extracted DNA was precipitated by ethanol and separated by centrifugation at 12000 rpm (at 4°C) for 15 min and the pellets were purified. The purified DNA was electrophorised using agarose gel electrophoresis at 150 V for about 90 min to assess the degree of apoptotic DNA fragmentation according to Gao *et al*., [23]. The DNA and its fragments on gel were detected by treatment with ethidium bromide and the florescence produced by UV light was photographed and analyzed using gel documentation system (G box, syngene, UK).

### Hypochromicity evaluation

This experiment was done to evaluate the possibility of active ingredients (A-I) to interchelate with DNA (isolated from EACC cell line), whose absorption decreases upon binding at 260 nm (term called hypochromicity). The comparison was done against free DNA and the tested compounds.

### Statistical analysis

Data were subjected to an analysis of variance, and the means were compared using the “Least Significant Difference (LSD)” test at 0.01 levels, as recommended by Snedecor and Cochran [24]. Data are presented as mean ± SD.

## Results and discussion

### Active ingredients

**Figure 1:**
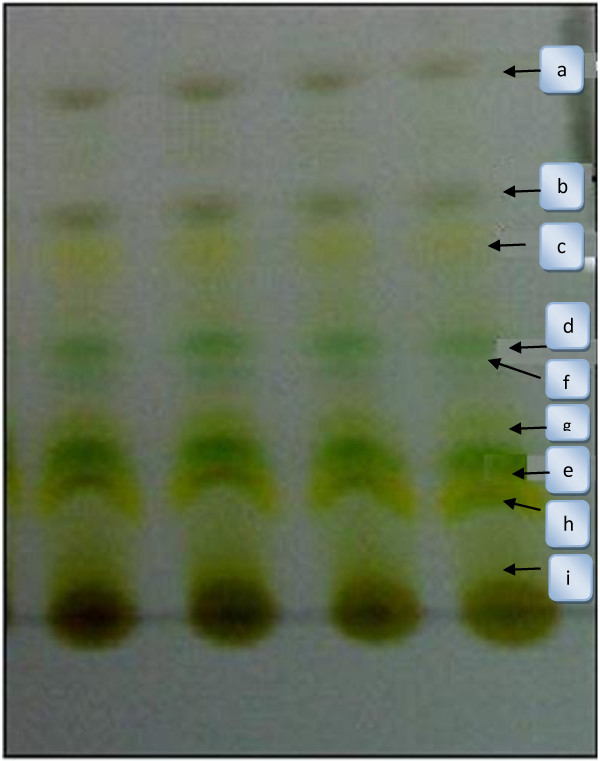
**Fractionation of crude methanolic extract of** ***Eichhornia crassipes*** **using silica gel TLC and hexane/ethyl acetate (8.5:1.5, v/v) as mobile phase.**

**Figure 2:**
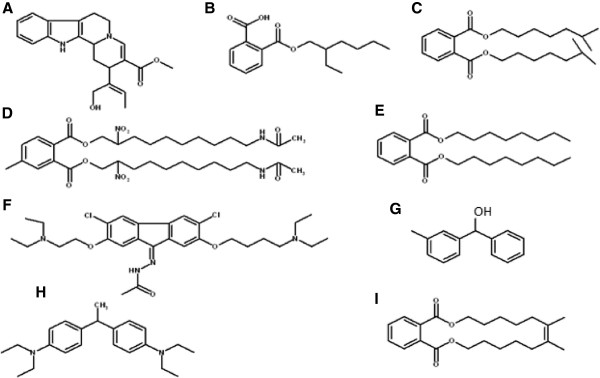
**Suggested chemical structure of active compounds separated from** ***Eichhornia crassipes.*** **A**: (18, 19-Secoyohimban-19-oic acid, 16, 17, 20, 21-tetradehydro-16-(hydroxymethyl)-, methyl ester (15 beta, 16 E) M. W. 352 Da **B**: 1,2-Benzene dicarboxylic acid, mono-(2-ethylhexyl ester) M. W 278 Da **C**: 1, 2 Benzene dicarboxylic acid, diisooctyl ester M. W. 390 Da. **D**: Di amino-di nitro-Methyl dioctyl phthalate M. W. 662 Da **E**: 1, 2 Benzene dicarboxylic acid, dioctyl ester M. W. 390 Da. **F**: 9-(2,2-Dimethyl propanoilhydrazono)-2,7-bis-[2-{diethylamino)-ethoxy] fluorine M.Wt=576. **G**: (3-Methyl phenyl)-phenyl methanol M.Wt.=198. **H**: 4- (diethylamino)-alpha-[4-(diethylamino) phenyl] M.Wt.=326. **I**: Isooctyl phthalate M. Wt= 390.

Nine fractions were separated and identified from water hyacinth crude extract (Figures 1 and 2) and showed biological activities as antioxidant and anticancer. The obtained data of the spectroscopic analysis revealed the nine compounds as; the alkaloid derivatives (A, D and F), terpenoids derivatives (B, C, E, G, H and I). The more or less comparable activities of the fractionated compounds from methanolic crude extract of *E. crassipes* may be due to the fact that all compounds have the common fragment ions: 57, 71, 85, 149, 167 Da and these results were confirmed by H^1^-NMR. The NMR data indicated that all compounds had the following type of protons; A multiplex signal at δ 6.89-7.47 ppm was characteristic of aromatic protons (Compounds A-I) according to Edwards [25]; While, the singlet signal at δ 5.320 ppm was characteristic of the two protons of olifinic (CH = CH) in compound A; the singlet signal at δ 3.342 ppm was characteristic of four protons of two O-CH_2_ group (Compounds B, C, D, E, F and I); the singlet signal at δ 1.26, 1.6 and 2.5 ppm was characteristic of the protons of methylene (CH_2_) group and the singlet signal at δ 1.9 ppm was characteristic of protons of methyl (CH_3_) group.

### Antioxidant activity

The DPPH scavenging assay was performed to test the antioxidant activity (as IC_50_) of both the crude methanolic extract and the nine TLC separated fractions (compounds) of *Eichhornia crassipes* (Table 1). The crude methanolic extract showed the highest antioxidant activity with IC_50_ of 74.80 ± 4.5 μg/mL. Three of nine compounds recorded more or less lower and comparable activities i.e. 92.40 ± 6.5, 95.4 ± 3.1 and 96.5 ± 2.7 μg/mL of compounds D, E and H. Additionally, compounds B, C, F, G and I were illustrated very close antioxidant effects with IC_50_ ranged between 97.0 ± 5.4 and 97.4 ± 2.7 μg/mL. However, compound A showed very low effect (99.2 ± 5.8 μg/mL) as antioxidant compared with synthetic (BHA and BHT) and natural (Vit. C) Standards.

**Table 1:** **IC**_**50**_ **(μg/mL) of different compounds separated from** ***Eichhornia crassipes*** **against DPPH and ABTS radical, and its relative percentage to Ascorbic acid as natural antioxidant**

Materials	DPPH method	ABTS method	Relative percentage to Vit. C
**Crude extract**	74.8 ± 4.5^f^	50.8 ± 2.4^g^	23.64
**Compound A**	99.2 ± 5.8^a^	77.0 ± 3.2^c^	15.6
**Compound B**	97.1 ± 3.9^b^	78.5 ± 2.4^a^	15.29
**Compound C**	97.4 ± 2.7^b^	77.6 ± 1.3^b^	15.47
**Compound D**	92.4 ± 6.5^e^	73.2 ± 4.2^e^	16.39
**Compound E**	95.4 ± 3.1^d^	75.8 ± 2.6^d^	15.84
**Compound F**	97.2 ± 4.6^b^	77.9 ± 1.9^ab^	15.40
**Compound G**	97.2 ± 1.6^b^	75.8 ± 1.6^d^	15.84
**Compound H**	96.5 ± 2.7^c^	69.8 ± 3.0^f^	17.20
**Compound I**	97.0 ± 5.4^b^	76.8 ± 3.1^c^	15.62
*BHT	8.7 ± 0.5^i^	5 ± 0.02^j^	
*BHA	11.5 ± 0.95^h^	9.4 ± 0.55^i^	
*Ascorbic acid	18.6 ± 1.0 g	12.0 ± 0.60^h^	
**LSD 0.01**	0.387	0.569	

*Standard antioxidants.

Each value is represented as mean of triple treatments, means within each row with different letters differ significantly at *P* < 0.01 according to Duncan’s multiple range test.

Activity was decreased on separation of the fractions in pure forms (compounds) and each more or less was lower than that of the crude extract. The potent antioxidant activity manifested by the crude extract in comparison to those of the separated compounds may be due to the synergistic action of the collective biologically active compounds of one or more of the nine fractions in the crude extract. In addition, the crude extract may have secondary metabolites in very low concentration which enhance the active principals and increase the antioxidant efficiency. This suggestion was previously confirmed by Chu *et al* [26] - Who mentioned that crude extract of *Spirulina* gave higher antioxidant activity than pure compounds (phycocyanin). They reported that the extract might contain other constituents (e.g. phenolic compounds) which gave a higher combined antioxidant activity than phycocyanin alone. The synergistic action of a wide spectrum of antioxidants may be more effective than the activity a single antioxidants.

It is difficult to give a definite explanation for mechanism of antioxidant of *Eichhornia* purified compound dependent on one assay. Thus, radical scavenging properties of the pure compounds (A-I) were evaluated against ABTS radical assay, and the results were compared with those of some standard antioxidant (BHT, BHA and Ascorbic acid) (Table 1). It was observed that those compounds showed a markedly higher ability to scavenge ABTS radicals than DPPH. The obtained data showed the trend concerning the crude extract as well as tested compounds. Crude extract scavenged the ABTS radical with IC_50_ values of 50.8 μg/mL. These values were better than the IC_50_ of DPPH radical (74.8 μg/mL) and other fractions but less than standard compounds (BHA, BHT and Ascorbic acid; 9.4, 5.0 and 12.0 μg/mL respectively). This means that, the compounds were strongly scavenged the ABTS radical than that of DPPH radical. These results are in agreement with those of Awika *et al*. [27] who found that, ABTS is a better choice than DPPH and more sensitive than DPPH. The ABTS method has the extra flexibility in that it can be used at different pH levels (unlike DPPH, which is sensitive to acidic pH) and thus is useful when studying the effect of pH on antioxidant activity of various compounds. It is also useful for measuring antioxidant activity of samples extracted in acidic solvents. Additionally, ABTS is soluble in aqueous and organic solvents and is thus useful in assessing antioxidant activity of samples in different media and is currently most commonly used in simulated serum ionic potential solution (pH 7.4 phosphate buffer containing 150 mM NaCl) (PBS). Another advantage of ABTS + method was that samples reacted rapidly with ABTS in the aqueous buffer solution (PBS) reaching a steady state within 30 min. The DPPH reacted very slowly with the samples, approaching, but not reaching, steady state after 8 h. This slow reaction was also observed when ABTS reacted with samples in alcohol.

The antioxidant activity of active ingredients separated from *E. crassipes* may be correlated with the presence of hydroxyl group and unsaturated bonds in the chemical structure of its compounds which show high ability for scavenging free radicals and prevent the oxidation processes. These observations were in agreement with the previously published results [28].

ROS are products of a normal cellular metabolism and play vital roles in the stimulation of signaling pathways in plant and animal cells in response to changes in intra- and extracellular environmental conditions [29]. Proteins, nucleic acids and lipids were also significant targets for oxidative attack, and modification of these molecules can increase the risk of mutagenesis [30]. Therefore, antioxidants are good scavengers for ROS and free radicals, on other word it defends and protect cells from their bad action. The antioxidants prevent damages of proteins, DNA (protect from mutation) and lipid peroxidation (protect plasma membrane) in living cells (normal cells).

### Antitumor activity

**Figure 3:**
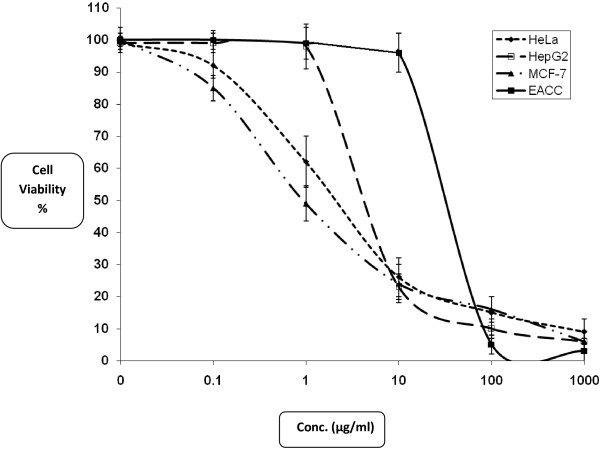
**Dose-response curve of** ***Eichhornia crassipes*** **crude extract in solid Tumor cell line culture of HeLa (●) HepG2 (○) MCF-7 (▼) and EACC (■) cells.** Cells were exposed to the crude extract for 72 h. Cell viability was determined by using of SRB-U assay while for EACC the incubation was for 4 h and viability was determined by use Trypan blue assay and data are expressed as mean ± S.D (n = 3).

SRB assay was used to assess the cytotoxicity of the crude extract and its derived fractions against three different solid tumor cell lines and one ascites tumor cell line. Different cell lines were used according to their origin and morphology as well as sensitivity and receptor sites behavior. The cytotoxicity parameter, IC_50_ was calculated using E_max_ model as described in the Methods section. Compound is considered strongly potent when IC_50_ is lower than 1 μg/mL; moderate if IC_50_ ranges from 1 μg/mL to 10 μg/mL; and weak for IC_50_ higher than 10 μg/mL. The obtained results of the crude extract showed acceptable potency against HeLa and MCF-7 cell lines with IC_50_ of 1.6 ± 0.5 and 1.2 ± 0.2 μg/mL, respectively. However, HepG2 and EACC cell lines showed relatively higher resistance against the crude extract with IC_50_ of 7.6 ± 1.5 and 6.04 ± 0.5 μg/mL respectively, when compared with Doxorubicin (DOX) as standard anticancer drug (with IC_50_ = 0.28, 0.42 and 0.42 μg/mL against Hela, HepG2 and MCF-7 respectively) as shown in Table 2. This means that the cytotoxicity pattern of the crude extract on both MCF-7 and HeLa cell lines is similar while differs on HepG2 and EACC (Figure 3). These results indicate that the effect of crude extract on MCF-7 and Hela cells is concentration dependant through the concentrations tested (1-1000 μg/mL). In HepG2 cells however, slight toxicity was noticed at low concentrations (below 10 μg/mL) after which the effect was very strong hence most of cells were died at about 15 μg/mL. This effect can be explained as receptor independent for these type of cells [31, 32]. Upon fractionation, compound I showed the most potent cytotoxic profile among all other fractions with IC_50_ of 0.8 ± 0.4 μg/mL (in HepG2 liver cancer cell line). Compound D showed also high potent cytotoxicity against HeLa cervix cancer cell line (IC_50_ = 4.3 ± 2.3 μg/mL) and the other compounds showed moderate cytotoxic effect with IC_50_’s ranging from 7.7 to 14.1 μg/mL. With respect to MCF-7 breast cancer cell line, compounds H, B and A showed the best cytotoxic profile with IC_50_’s of 11.1 ± 6.1 μg/mL, 13.4 ± 1.9 μg/mL and 13.6 ± 5.3 μg/mL, respectively. In addition, the other compounds G and C showed mild cytotoxic effects with IC_50_’s ranging from 17.5 to 69.1 μg/mL. The other compounds showed much humble cytotoxicity profile against HepG2 cell line with IC_50_’s ranging from 14.9 to 74.2 μg/mL. Concerning EACC cancer cell line, compounds D, C, E, A showed high potency with IC_50_ of 6.42 ± 0.8, 7.29 ± 1.6, 8.19 ± 1.2 and 8.61 ± 2.1 μg/mL, respectively (Table 2). The other compounds showed moderate effect with IC_50_ of 12.32 ± 2.7, 12.67 ± 4.2 and 22.79 ± 6.9 μg/mL for compounds H, G and I, respectively. The effect of plant extract and its compounds on EACC is very similar to their effects on HeLa cells (Table 2).

**Table 2:** **Cytotoxicity [Expressed as IC**_**50**_ **(μg/mL)] of crude extract of** ***Eichhornia crassipes*** **and its fractionated products on different tumor cell lines**

Materials	IC _50_(μg/mL)			
	HeLa cell line	HepG2 cell line	MCF-7 cell line	EACC
**Crude extract**	1.6 ± 0.5^j^	7.6 ± 1.5^i^	1.2 ± 0.2^i^	6.04 ± 0.5^j^
**Compound A**	7.7 ± 4^g^	40.2 ± 10.1^c^	13.6 ± 5.3^g^	8.61 ± 2.1^f^
**Compound B**	10.7 ± 1.3^d^	28.3 ± 3.7^d^	13.4 ± 1.9^g^	17.3 ± 3.5^b^
**Compound C**	12.8 ± 5.1^b^	74.2 ± 12.5^a^	19.4 ± 9.2e	7.29 ± 1.6^h^
**Compound D**	4.3 ± 2.3^i^	23.6 ± 7^f^	27.2 ± 2.5^d^	6.42 ± 0.8^i^
**Compound E**	9.9 ± 3.4^e^	56.1 ± 12.3^b^	31.4 ± 9.6^c^	8.19 ± 1.2^g^
**Compound F**	6.9 ± 3.1^h^	14.9 ± 8.1^h^	41.3 ± 6.4^b^	9.9 ± 2.6^e^
**Compound G**	9.0 ± 3.7^f^	23.8 ± 5.4^e^	17.5 ± 4.7^f^	12.7 ± 4.2^c^
**Compound H**	14.1 ± 7.0^a^	15.4 ± 3.6^g^	11.1 ± 6.1^h^	12.3 ± 2.7^d^
**Compound I**	11.8 ± 7.0^c^	0.8 ± 0.4^j^	69.1 ± 4.9^a^	22.8 ± 6.9^a^
*DOX	0.28 ± 0.07^j^	0.42 ± 0.05^j^	0.42 ± 0.11^j^	--
**LSD 0.01**	0.225	0.16	0.284	0.188

Each value is presented as mean of triple treatments, means within each row with different letters differ significantly at P < 0.01 according to Duncan’s multiple range test.

*DOX: Doxorubicin (Standard anticancer drug).

It’s worth mentioning, that the higher potency of the crude extract against cancer cell lines in special HeLa and MCF-7 relative to all fractions (compounds) from the same extract, might be attributed to auto-synergistic effect of these fractions within the same extract. In addition, the crude extract may have additional secondary metabolites in very low concentration which may enhance the active principals and increase the anticancer efficiency in addition to its stability against enzyme action of tumor cells as mentioned in antioxidant activity [26].

The similar cytotoxic profile of the crude extract against both HeLa (cervix cancer) and MCF-7 (breast cancer) cell lines followed by EACC might be attributed to their gynecological origin and hormone dependency [33]. Several gynecological solid tumors such as breast and cervix cancers are greatly influenced by hormone level and signaling within tumor micro *milieu* [34, 35]. Further studies are recommended to identify potential interference between the isolated potent fractions and estrogenic signaling pathway within gynecological tumors.

**Figure 4:**
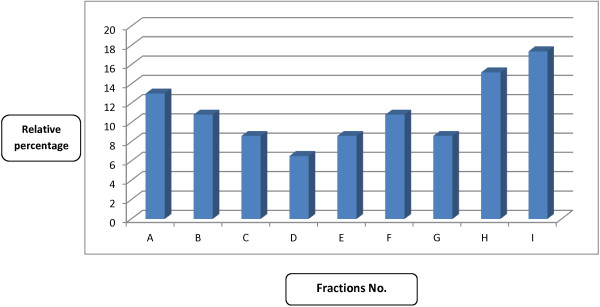
**Relative percentage (%) of different fractions separated from methanolic extract of** ***Eichhornia crassipes*** **.**

In liver cancer cell line, HepG2, the only compound with promising potency was compound I, identified as iso-octyl phthalate (IC_50_ = 0.8 ± 0.4 μg/mL). Similar trend was noticed for HeLa and EACC cell lines hence, compound D (methyl dioctyl phthalate) showed high potency as anticancer. This information recommends further SAR and QSAR studies to identify the exact target receptor for these active compounds. Herein, it is interesting to notice that most of the detected phthalate esters have a high potency against cancer cell lines. The longest side chain-ester of phthalate (compound D) has the strongest anticancer activity against HeLa and EACC cell lines. In this concern, Yamamoto *et al*. [36] reported that phthalate derivatives (compound B, C, D, E and I) had high ability for inhibition of skin tumor promotion induced by 12-O-tetradecanyl-phorbol-13-acetate. In addition, the present data showed that compound D presented the lowest percentage in the crude extract (only 6%, Figure 4) but has the highest antioxidant and anticancer activity. In contrast, compound I represented the highest percentage fraction (more than 17%) in the crude extract (Figure 4) and showed the strongest anticancer activity against Hep-G2 and EACC respectively (Table 2).

**Table 3:** **Hypohromicity and Hyperchromicity of active ingredients separated from** ***Eichhornia crassipes***

Compounds		DNA	Compounds + DNA		
	O.D	O.D	Expected	Observed	
	260 nm	260 nm		After 2 h	After 24 h
**A**	0.0325	0.6995	0.732	0.289	0.391
**B**	0.139		0.838	1.032	1.196
**C**	0.065		0.764	0.893	1.168
**D**	0.0995		0.7985	0.906	1.042
**E**	0.0725		0.7715	0.863	0.99
**F**	0.3085		1.0075	1.036	1.050
**G**	0.1245		0.8235	0.956	1.07
**H**	0.1905		0.8895	1.080	1.04
**I**	0.0850		0.7840	0.841	0.928

**Figure 5:**
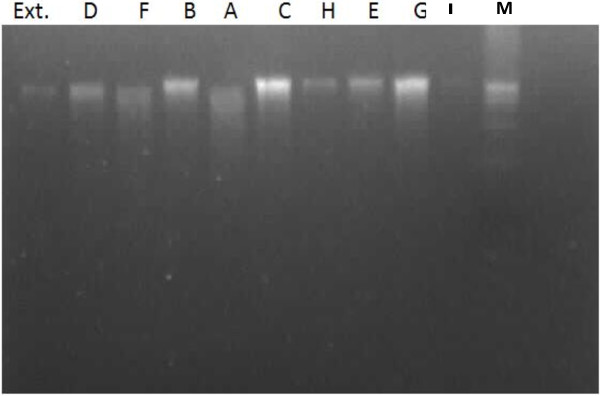
**DNA fragmentation patterns obtained after incubation of tested compounds (A-I) with different tumor cells.** (M: DNA marker).

The anticancer activities of compounds A, G and H were not recorded in any other investigation concerned with neither *Eichhornia crassipes* nor other plants (its first record in this study). The later two compounds (G and H) seem to have potent activity for the four cancer cell lines tested in spite of the fact that they came in the second order of anticancer activity. These compounds constitute only about 22.5% (relative) of the crude extract (G 8% and H 14.5%, Figure 4) which may be more potent by increasing their contents in the media. Therefore, experiment was done to confirm this probability hence, incubation of cancer cells with higher concentrations of each compounds showed higher anticancer activity (EACC was used, data not shown). Also using mixture of the two compounds gave the most potency. In spite of occurring compound G in crude extract in lower percentage but it showed high anticancer activity for the four cell lines used. In this respect, the two compounds are very similar in their chemical structure (formulas), hence they contain biphenyl linked through methylene group and the later carries methyl group. This structure may enhance liberation of methyl ion as carbonium ion which affects DNA of cancer cells [37] or cell proteins and/or enzymes. This liberated methyl ion group can bind with N7- guanine base (or other bases) in DNA and induce mutation which in turn stop cell life cycle or kill these cancer cells [38]. This phenomenon resulted in significant intensities of internucleosomal DNA fragmentations, evidenced by the formation of a DNA laddering on agarose gel (Figure 5), hallmark of cells undergoing apoptosis. This means that nuclear DNA was degraded to different fragments of about 200 bp or its folds which appeared as ladder. This appeared clearly by crude extract treatments and compound I followed by compounds H and A and then F and E (lanes 10, 7, 5, 3 and 8 respectively, Figure 5).

The presence of short side chain in compound H (but not compound G) may enhance methyl ion liberation due to their electro negativity potential. The potency of H compound was expected, which showed higher anticancer activity than compound G as noticed in Table 2. Data of the present study also showed that, HeLa cells and EACC have higher sensitivity to natural anticancer (extracts or compounds) than HepG2 and MCF-7 cell lines which showed resistant behavior for the treatments. In this concern, we found that the incubation of these cells with mixture of the high potent compounds (I, F, G and D) gave high anticancer activity and the anticancer effect was concentration dependent.

**Figure 6:**
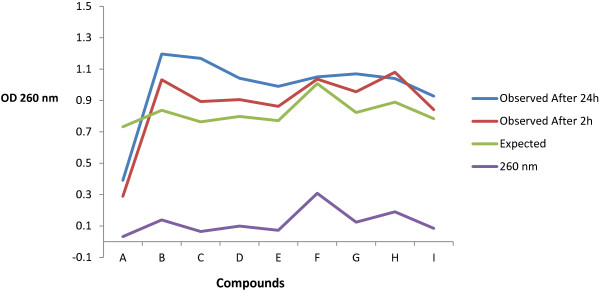
**Hypochromicity of active compounds separated from** ***Eichhornia crassipes.***

We did an additional experiment to test the interchelation possibility of each compound with DNA of the cancer cells (detection of hypochromicity through absorption at 260 nm). Results in Table 3 and Figure 6 showed that compound A was the only compound which can interchelate with DNA while the other eight compounds (B-I) were not. The absorbance of DNA + compound mixture was increased (loss of hypochromecity) after 2 and 24 h of mixture which illustrated DNA damage. Therefore, the damage in cancer cells by specific natural compounds did not depend only on their interchelation with DNA but might also to strands cleavage (DNA denaturation) or the base damages [by alkylation and/or free radical attack] or its liberation.

In this concern, we conclude that natural anticancer substances can affect cancer cells through their DNA damage by different probability mechanisms.

### Antioxidant-anticancer relationship

The present work showed a relationship between the antioxidant activity of natural compounds and their anticancer potency.

In this concern, extensive research during the past 2 decades has revealed the mechanism by which continued oxidative stress can lead to chronic inflammation, which in turn could mediate most chronic diseases including cancer, diabetes, and cardiovascular, neurological, and pulmonary diseases. Oxidative stress can activate a variety of transcription factors including NF-κB, AP-1, p53, HIF-1α, PPAR-γ, β-catenin/Wnt, and Nrf2. Activation of these transcription factors can lead to the expression of over 500 different genes, including those of growth factors, inflammatory cytokines, chemokines, cell cycle regulatory molecules, and anti-inflammatory molecules [38].

On the other side, the role of antioxidant compounds on growth inhibition of cancer cells could be explained as one or more of following suggested mechanisms:

1. The resonance phenomena of double bonds and lone pair atoms (N, S, O) in the chemical structure of the active compound (Figure 2). This structure may lead to radical formation in more than one site; for example: benzene ring (A), this ring is near to highly negativity group (nitro group); this condition helps the benzene ring to convert it to radical formation of new covalent bond with ABTS radical (as antioxidant activity). This radical may react with nitrogen bases of DNA and lead to mutation in nucleic acid of cancer cells and affect cell division (acts as anticancer activity) [39].
2. Normal tissues typically have median oxygen concentrations in the range 40–60 mmHg, half of all solid tumors have median values less than 10 mmHg with fewer than 10% in the normal range. So, the antioxidant compounds may be affecting on cancer cells by providing high concentration of oxygen by acting with its radicals form and in turn disturbs tumor hypoxia needed for these cells [35].
3. Recent studies have demonstrated that antioxidant compounds (including selenium) are also essential elements of redox system, which has been shown to have multiple functions, including regulating cell growth and apoptosis [2, 3]. Selenium may exert its effects in cancer cells by altering intracellular redox state, which subsequently results in cell cycle block. In addition, these compounds have been demonstrated to possess antitumorigenic activities in animal models by inhibiting tumor initiation and promotion through disturbing redox potential system of tumor cells. This takes place *via* modifying cellular antioxidants and antioxidant enzymes, thus regulating the therapeutic effectiveness of natural drugs in cancer therapy [40].
4. As recommendation, the addition of antioxidant compounds with anticancer chemical drug enhances the action of chemotherapies. In this concern, an antiestrogen drug skeleton was found to induce cytotoxicity towards breast-cancer cells that are resistant to the common antiestrogen drug. The efficiency of this drug has been related to the proton-coupled electron transfer observed in the presence of pyridine as a base [41].

## Conclusion

It can be concluded that the pronounced results may encourage a country-wide project for not only collecting and getting rid of water Hyacinth but also making a pharmaco-economic value in Egypt. Harvesting water hyacinth, not only clean the drinking water from its deleterious effect but also would be used for the production of pharmaceutical remedies. In addition, they have scavenging antioxidant properties against the reactive oxygen species. The present study reveals the high potency of crude extracts as antioxidants and potential anticancer in comparison to their separated fractions. This crude extract represents favorable economic and industrial value for the production of commercial product. The pure compound separately or combined with other compound(s) or/and crude extract could be used as natural antioxidant and anticancer formulas.

## References

[1] Moerman DE (1996). An analysis of the food plants and drug plants of native North America. J Ethnopharmacol.

[2] Dev S (2000). Impact of natural products in modern drug development. Indian J Exp Biol.

[3] Haroon AM (2006). Effect of some macrophytes extracts on growth of *Aspergillus parasiticus*. Egyptian J Aquatic Res.

[4] Ndubuisi JA, Emeka OE, Luke UN (2007). Physicochemical properties of choloform extract of water hyacinth (Eichhornia crassipes). Afr J Plant Sci Biotech.

[5] Ghoshal S, Prasad BN, Lakshmi V (1996). Antiamoebic activity of Piper longum fruits against *Entamoeba histolytica in vitro* and *in vivo*. J Ethnopharmacol.

[6] Akinmoladun AC, Obuotor EM, Farombi EO (2010). Evaluation of antioxidant and free radical scavenging capacities of some Nigerian indigenous medicinal plants. J Med Food.

[7] Stevens JF, Hart HT, Hendriks H, Malingre TM (1992). Alkaloids of some European and macaronesian sediodege and semepervivodeae (crassulaceae). Phytochemical.

[8] Khattab FA: *The problem of water hyacinth**Eichhornia crassipes**in Egypt and methods of management.* Lagos: Proceedings of the International Workshop/seminar on water hyacinth;

[9] HEA A (2008). MSC Thesis. Ecology and Adaptation of Water Hyacinth in the Nile Delta Ecosystem.

[10] Dandelot S, Robles C, Pech N, Cazaubon A, Verlaque R (2008). Allelopathic potential of two invasive alien Ludwigia spp. Aquat Bot.

[11] Baublis AJ, Lu C, Clydesdale FM, Decker EA (2000). Potential of wheat-based breakfast cereals as a source of dietary antioxidants. J Am Coll Nutr.

[12] Halliwell B (1996). Oxidative stress, nutrition and health. Experimental strategies for optimization of nutritional antioxidant intake in humans. Free Radic Res.

[13] Yu L, Haley S, Perret J, Harris M, Wilson J (2002). Free radical scavenging properties of wheat extracts. J Agric Food Chem.

[14] Yu L, Perret J, Harris M, Wilson J, Haley S (2003). Antioxidant properties of bran extracts from “Akron” wheat grown at different locations. J Agric Food Chem.

[15] Sudjaroen Y, Haubner R, Wurtele G, Hull WE, Erben G (2005). Isolation and structure elucidation of phenolic antioxidants from Tamarind (Tamarindus indica L.) seeds and pericarp. Food Chem Toxicol.

[16] Lata N, Dubey V (2010). Quantification and identification of alkaloids of Eichhornia crassipes: the world’s worst aquatic plant. J Phar Res.

[17] Shanab SM, Shalaby EA, Lightfoot DA, El-Shemy HA (2010). Allelopathic effects of water hyacinth [Eichhornia crassipes]. PLoS One.

[18] Burits M, Bucar F (2010). Antioxidant activity of *Nigella sativa* essential oil. Phytother Res.

[19] Re R, Pellegrini N, Proteggente A, Pannala A, Yang M (1999). Antioxidant activity applying an improved ABTS radical cation decolorization assay. Free Radic Biol Med.

[20] Freshney RI (2002). Cell line provenance. Cytotechnology.

[21] Skehan P, Storeng R, Scudiero D, Monks A, McMahon J (1990). New colorimetric cytotoxicity assay for anticancer-drug screening. J Natl Cancer Inst.

[22] Retpetto G, Peso A, Zurita J (2008). Neutral red uptake assay for the estimation of cell viability/cytotoxicity. Nat Protoc.

[23] Goa X, Xu Y, Divine G, Janakirman W, Champan R (2002). Disparate *in vitro* and *in vivo* antileukemic effects of resveratrol, a natural polyphenolic compound found in grapes. J Nutr.

[24] Snedecor GW,, Cochran WG (1982). Statistical Methods.

[25] Edwards JC: **Principles of NMR.** *Process NMR Associates LLC* 87A Sand Pit Rd, Danbury CT 06810, February 2009. http://www.process-nmr.com/Edwards/John%20Edwards%20-%20CV.pdf

[26] Chu W, Lim Y, Radhakrishnan AK, Lim P (2010). Protective effect of aqueous extract from *Spirulina platensis* against cell death induced by free radicals. BMC Complement Altern Med.

[27] Awika JM, Rooney LW, Ronald XW, Prior L, Cisneros-Zevallos L (2003). Screening methods to measure antioxidant activity of Sorghum (*Sorghum bicolor*) and Sorghum products. J Agric Food Chem.

[28] Michalak A (2006). Phenolic compounds and their antioxidant activity in plants growing under heavy metal stress. Pol J Environ Stud.

[29] Jabs T (1999). Reactive oxygen intermediates as mediators of programmed cell death in plants and animals. Biochem Pharmacol.

[30] Schraufstatter I, Hyslop PA, Jackson JH, Cochrane CG (1988). Oxidant-induced DNA damage of target cells. J Clin Invest.

[31] Brown JM (2000). Exploiting the hypoxic cancer cell: mechanisms and therapeutic strategies. Mol Med Today.

[32] Westerink WMA, Schoonen WGAJ (2007). Cytochrome P450 enzyme levels in HepG2 cells and cryopreserved primary human hepatocytes and their induction in HepG2 cells. Toxicol In Vitro.

[33] Ruiz-Cabello J, Berghmans K, Kaplan O, Lippman ME, Clarke R (1995). Hormone dependence of breast cancer cells and the effects of tamoxifen and estrogen: 31P NMR studies. Breast Cancer Res Treat.

[34] Ndebele K, Graham B, Tchounwou PB (2010). Estrogenic activity of coumestrol, DDT, and TCDD in human cervical cancer cells. Int J Environ Res Public Health.

[35] Van der Burg B (1991). Sex steroids and growth factors in mammary cancer. Acta Endocrinol (Copenh).

[36] Yamamoto S, Nakadate T, Aizu E (1990). Anti-tumor promoting action of phthalic acid mono-nbutyl ester cupric salt, a biomimetic superoxide dismutase. Carcinogenesis.

[37] Lundin C, North M, Erixon K, Walters K, Jenssen D, Goldman ASH, Helleday T (2005). “Methyl methanesulfonate (MMS) produces heat-labile DNA damage but no detectable in vivo DNA double-strand breaks”. Nucleic Acids Res.

[38] Reuter S, Gupta Madan CS, Chaturvedi M, Aggarwal BB (2010). Oxidative stress, inflammation, and cancer: How are they linked?. Free Rad Biol Med.

[39] Shanab SMM, Shalaby EA, El-Fayoumy EA (2011). E*nteromorpha compressa* exhibits potent antioxidant activity. J Biomed Biotechnol.

[40] Zhong W, Oberley DW (2001). Redox-mediated effects of selenium on apoptosis and cell cycle in the LNCaP human prostate cancer cell line. Cancer Res.

[41] Hillard E, Vessières A, Thouin L, Jaouen G, Amatore C (2005). Ferrocene-mediated proton-coupled electron transfer in a series of ferrocifen-type breast-cancer drug candidates. Angew Chem Int Ed Engl.

[CR42] The pre-publication history for this paper can be accessed here:http://www.biomedcentral.com/1472-6882/14/397/prepub

